# Effectiveness and implementation of a multidisciplinary lifestyle focused approach in the treatment of inpatients with mental illness (MULTI +): a stepped wedge study protocol

**DOI:** 10.1186/s12888-022-03801-w

**Published:** 2022-03-31

**Authors:** N. M. den Bleijker, M. M. E. van Schothorst, I. J. M. Hendriksen, W. Cahn, N. K. de Vries, P. N. van Harten, J. Deenik

**Affiliations:** 1 Scientific Research Department, GGz Centraal, Amersfoort, the Netherlands; 2 Department of Psychiatry, UMC Utrecht Brain Center, University Medical Center Utrecht, Utrecht University, Utrecht, the Netherlands; 3 School for Mental Health and Neuroscience, Faculty of Health, Medicine and Life Sciences, Maastricht University, Maastricht, the Netherlands; 4 LivIng Active, Santpoort-Zuid, the Netherlands; 5 Faculty of Health, Medicine and Life Sciences, Maastricht University, Maastricht, the Netherlands

**Keywords:** Mental illness, Lifestyle, Inpatients, Effectiveness, Implementation

## Abstract

**Background:**

People with mental illness have a reduced life expectancy compared to the general population. Despite the increasing evidence for the efficacy of lifestyle interventions there is little change in routine clinical care. This discrepancy is often referred to as the implementation gap and has caused a need for effectiveness and implementation research in real-world settings. Our study assesses the effectiveness and implementation of a multidisciplinary lifestyle focused approach in the treatment of inpatients with mental illness (MULTI +).

**Methods:**

An open cohort stepped wedge cluster randomized trial in inpatients psychiatric wards of GGz Centraal, the Netherlands. The wards are divided into three clusters based on geographical region. These clusters are randomly allocated to one of the three pre-defined steps to integrate MULTI + . MULTI + can be tailored to fit individual psychiatric wards and includes 10 core components aimed at improving lifestyle factors. The primary outcome is to investigate the difference in the mean QRISK3 score of patients receiving MULTI + compared to patients receiving TAU. Secondary outcomes include somatic and mental health outcomes, lifestyle factors, and implementation factors. Findings will be analysed using mixed model analyses.

**Discussion:**

The MULTI + study is the first large-scale study evaluating the long-term effects of a multidisciplinary, multicomponent approach aimed at improving lifestyle factors in routine inpatient mental health care. A limitation of this study is the risk of missing data due to the large-scale, real-world setting of this study. Furthermore, implementation monitoring and external events that may influence outcomes could be difficult to account for. Strengths of this study are the focus on effectiveness as well as implementation and the inclusion of both patient and health care professionals’ perspectives. Effectiveness studies in routine clinical care can advance our knowledge on lifestyle interventions in real-world settings.

**Trial registration:**

ClinicalTrials.gov registration. Identifier: NCT04922749. Retrospectively registered 3th of June 2021.

**Supplementary Information:**

The online version contains supplementary material available at 10.1186/s12888-022-03801-w.

## Background

People with mental illness (MI) have a reduced life expectancy compared to the general population, mostly attributable to somatic diseases caused by poor physical health [1, 2]. Irrespective of their diagnosis, people with MI have 1.4–2 times higher risk of cardiometabolic diseases (e.g. cardiovascular disease (CVD) and diabetes), many of which can potentially be avoided [3–5]. Modifiable lifestyle factors such as physical activity (PA), diet, sleep and smoking behaviour have been increasingly associated with the onset of somatic diseases in people with MI [3, 5–8]. Adverse health behaviours are prevalent in people with MI and the resulting poor physical health is often exacerbated by the adverse effects of psychotropic medication [9, 10]. Hence, it is important to stimulate healthy lifestyle behaviours in people with MI.

There is increasing clinical and scientific evidence for the efficacy of interventions targeting lifestyle factors [6]. A series of reviews and meta-analyses that analysed interventions aimed at improving PA [11, 12], nutrition and diet [13, 14], sleep [15, 16], and smoking cessation [17] have shown favourable results on cardiometabolic health, psychosocial functioning and quality of life. In addition to this, reductions in MI-related symptoms such as negative symptoms, depression and psychotic symptoms were found. Despite this overwhelming evidence demonstrating the efficacy of interventions aimed at improving lifestyle factors, there have not been many structural changes in routine clinical care for people with MI [5, 18–21].

Implementing and sustaining interventions is an intricate process, and multiple factors can hinder the translation of evidence into practice [20, 22, 23]. For example, efficacy research mostly uses a randomized-controlled trial (RCT) design which can often not be translated linearly into routine clinical care, resulting in diminished effects [24, 25]. Moreover, trials often do not pass research stages, or are less likely to be sustained [22]. Pragmatic clinical trials could promote the implementation and sustainability into routine clinical care because they better reflect real-world conditions [26]. In addition, implementation factors, such as organisational culture and financial sustainment, can complicate the uptake and continuation of lifestyle interventions [27]. Studies investigating implementation factors are scarce, whilst several publications highlight their importance [6, 20, 28, 29]. Consequently, the focus of the present research is on the implementation gap (*i.e. how to make it work)*, rather than a knowledge gap (*i.e. does it work?)* [20].

The most efficacious lifestyle interventions are delivered by qualified health care professionals (HCPs) and target multiple lifestyle factors simultaneously [5, 29, 30]. However, research on such multidisciplinary, multicomponent lifestyle interventions is scarce and little is known about long-term effects and sustainability. Moreover, most research is conducted in outpatient settings. Further research in inpatient settings is especially important considering the high illness severity and comorbidity in this patient population [31].

Deenik and colleagues [30] previously evaluated a multidisciplinary, multicomponent approach (called MULTI) aimed at a holistic lifestyle change in a real-world inpatient setting for people with severe mental illness (SMI). They found positive changes in both somatic and mental health after 18 months compared to usual care, such as improvements in metabolic risk factors, psychosocial functioning and a reduction in the use of psychotropic medication [32, 33]. The authors urged to confirm and complement findings in scaled-up studies and made several suggestions for improvement of the approach and pragmatic research of implementation [30]. In line with these recommendations, MULTI has been further developed into MULTI + and will now be implemented on a larger scale. We aim to investigate the effectiveness and implementation of this multidisciplinary lifestyle focused approach in the treatment of inpatients with mental illness (MULTI +).

## Methods

### Study design and setting

This study is an open cohort stepped wedge cluster randomized trial conducted at all inpatient wards of the specialist mental healthcare organisation GGz Centraal (the Netherlands) [34]. The organisation will implement MULTI + semi-annually in three geographical regions (clusters). Wards are almost equally distributed per cluster, covering a total of ~ 830 places of residence in which ~ 2000 patients are treated annually (Table 1). This gradual implementation intends that all clusters are exposed to MULTI + at the end of this trial. Each step includes a one-month transitional phase to prepare the cluster for the implementation of MULTI + . This trial takes 18 months to complete.

**Table 1:** *Places of residence divided in three clusters*

**Cluster**	**Wards (n)**	**Places of residence (n)**
1	15	~ 300
2	14	~ 300
3	15	~ 230

In line with the regional implementation, cluster-level randomisation is used. Individual-level randomisation is not possible because patients cohabit in groups within wards. Moreover, cluster-level randomisation minimizes the risk for contamination between HCPs working in multiple wards or different geographical clusters. Due to high patient turnover the patient population may differ at each measurement and individual follow-up for long-term evaluation is not feasible after discharge.

**Fig. 1:**
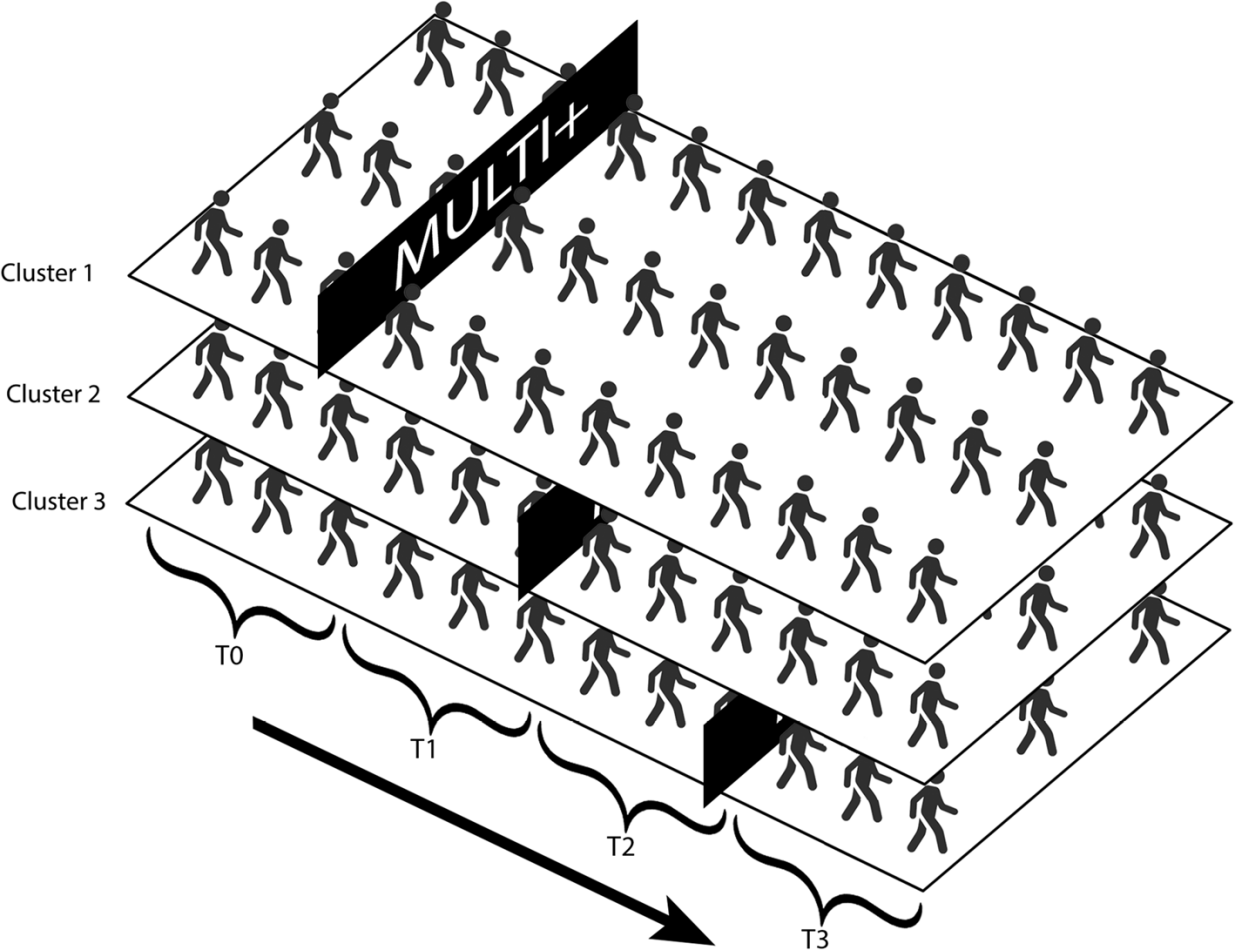
Open cohort stepped wedge cluster randomized trial in inpatient psychiatric wards in three clusters, demarcated by the dates of implementation of MULTI + . Measurements are obtained semi-annually

Measurements are obtained at the same time in all clusters at baseline (T0), after six months (T1), after twelve months (T2) and after eighteen months (T3). Measurements take approximately three months to complete (Fig. 1).

### Eligibility criteria

Patients aged ≥ 16 years who receive care at the inpatient psychiatric wards of GGz Centraal are eligible to participate in the study. Patients are not eligible to participate when they have limited knowledge or understanding of the Dutch language, or if their psychiatric or physical condition hinders informed consent at the discretion of the relevant physician, nurses, or researcher.

### Intervention

MULTI + is a multidisciplinary, multicomponent intervention which aims to improve lifestyle factors through a holistic approach. MULTI + focuses on 10 core components (i.e. the elements and activities that are necessary to achieve desired outcomes) [35], based on previous research and recommendations (Table 2) [5, 14, 30]. MULTI + is embedded in the long-term policy of the mental health care organisation, creating a support base and resources to facilitate implementation at all organisational levels. The implementation of the core components is co-designed with, and tailored to the ward and patient population, because of the large heterogeneity in patient characteristics and varying access to facilities and staffing.

**Table 2:** *10 core components of MULTI + and application into routine clinical care*

Core components of MULTI + ^a^	Application of core components
Routine daily structure and sleep	Wards offer a routine structure in a day-to-day program which includes standard mealtimes, sports-related activities, work-related activities, psychoeducation, and skills training. HCPs pay attention to the circadian rhythm and sleep hygiene of patients. It is intended that patients participate in at least two activities per day, such as walking in the morning and psychoeducation in the afternoon. HCPs motivate patients to engage in activities
Physical activity	Decreasing sedentary behaviour and increasing PA with the credo: “doing some physical activity is better than doing none” as a starting point. The eventual aim is to meet the (inter)national physical activity guidelines of ≥ 150 min of moderate-intensity PA every week, spread over several days [36, 37]. Additionally, patients engage in muscle- and bone-strengthening activities at least twice a week. Elderly patients combine these with balance exercises
Attention to nutrition and eating habits	Attention for the nutritional value and composition of meals and snacks in compliance with the Dutch national guidelines [38], as well as eating habits such as mealtimes and mindful eating. There are (preventive) consultations with dietitians on (clinical) indication of patients or HCPs
Substance use	Smoking cessation is encouraged in both HCPs and patients, and patients are supported by smoking interventions on (clinical) indication of patients or HPCs. For other substance use, patients are referred to addiction specialists
Multidisciplinary approach	Various disciplines are involved in the guidance of a single patient based on the patients’ needs and wishes, such as practitioners, nurses, exercise professionals and dietitians. These HCPs discuss the progress and compliance of individual patients in multidisciplinary consultations led by the chief physicians
Skills training	Daily living skills training and activities for patients, such as making a grocery list, shopping and cooking are embedded in the day-to-day program. Evidence-based behavioural techniques such as goal setting, planning and use of rewards are applied
Psychoeducation	Psychoeducation for patients, such as information about side-effects of medication, oral hygiene, sleep hygiene or nutrition is offered and embedded in the day-to-day program
Critical review of obesogenic environment and existing policies	HCPs examine the (obesogenic) environment and existing policies such as the availability of (un)healthy food and beverages, the use of personal transport and designated smoking areas, and adjust these if necessary. Behavioural change techniques such as choice-architecture and nudging are used
Active participation of HCPs	It is intended that at least one HCP participates in activities of the day-to-day program. HCPs do not engage in adverse health behaviours around patients, such as smoking and unhealthy eating as they have an exemplary function. HCPs motivate patients to improve their lifestyle factors
Training of HCPs	HCPs make use of the available educational resources and follow courses and training such as motivational interviewing or moral deliberation. These activities can support HCPs in motivating and educating patients, and facilitates awareness and culture change

*HCPs* Healthcare Professionals, *PA* Physical Activity

^a^Cross-compliance for implementation of core elements: culture change, peer support, co-designing, tailoring

MULTI + is implemented gradually in every cluster at ward level between November 2020 and November 2021. The explicit aim is the integration into routine clinical care instead of MULTI + being a temporary intervention. The chief physicians supervise the implementation. Four to six months before the start of implementation we approach the chief physicians to discuss the practical start with, and integration of the core components.

Because the organizational structure differs between clusters and wards, the practical dissemination and implementation of the core components of MULTI + may be adjusted where needed to promote sustainability. Nonetheless, every ward should have a multicomponent approach and have multidisciplinary consultations with qualified HCPs of relevant disciplines, such as practitioners, nurses, movement therapists and dietitians. HCPs develop active day-to-day programs in multidisciplinary work sessions tailored to their respective psychiatric wards. This day-to-day program includes various activities in accordance with the core components.

It is intended that patients participate in at least two activities per day to decrease sedentary behaviour. HCPs actively participate in these activities. Attending activities together contributes to joint responsibility and group feeling, increases peer support among patients, and facilitates culture change. When possible, patients are involved in designing the activities in the day-to-day program by fitting the activities to their capabilities and interests, to increase their intrinsic motivation. Compliance of patients with the intervention is discussed in multidisciplinary consultation among HCPs and extra support to the patient is provided when needed. The possibilities for co-design and tailoring makes MULTI + uniquely applicable to various forms of daily clinical care.

To establish long-term sustainability, an implementation team is formed to evaluate and safeguard ongoing improvement of MULTI + . This team consists of a senior researcher, a project leader and two junior researchers, and facilitates key activities in the implementation, improvement and continuation of MULTI + [39]. In collaboration with the implementation team, educational resources for HCPs and patients about lifestyle factors are developed to increase knowledge and enhance skills and capabilities. These resources are distributed using company-specific communication channels (such as internal webpages, e-mail, and teams’ pages), presentations, podcasts, e-learning modules, and webinars. The implementation team works in close collaboration with key stakeholders such as the board of directors, head physicians, nurse advisory board, the client association, and representatives of the family board. Research has shown that implementation teams can guide implementation and ensure faster, more successful outcomes [39].

### Treatment as usual (TAU)

TAU consists of pharmacological treatment, psychotherapy, and a less structured day-to-day program. Concomitant forms of care may be provided during the study.

### Data collection and management

Data is obtained from routine screening (from electronic patient files according to the organisational data protocol) and questionnaires. Questionnaire data, including demographics, is collected with Castor EDC [40]. This is a cloud-based electronic research data management platform for the secure collection and management of data. Questionnaires for HCPs are conducted through online surveys. We administer the questionnaires for patients through semi-structured interviews, because inpatients with MI often experience cognitive deficits, which can lead to impaired concentration, memory deficits and poor understanding of questions [41, 42]. Semi-structured interviews allow for the clarification of questions when needed. Due to illness severity and cognitive deficits, not all patients may be able to complete all questionnaires. Patients are included in this study if they complete at least one questionnaire. Conducting all questionnaires takes 45–60 min per patient, which can be completed in multiple appointments if necessary. A researcher or trained research assistant conducts the semi-structured interview. Training consists of a presentation of the research protocol and instructions on each questionnaire followed by role-play. To increase reliability, researchers follow an interview protocol. Weekly meetings are organized with the research team to discuss possible ambiguous answers.

### Primary outcome

The primary outcome of this study is to estimate the difference in the mean QRISK3 score of patients receiving MULTI + compared to patients receiving TAU. QRISK3 is an algorithm that estimates the probability of developing cardiovascular disease over the next 10 years [43].

### Secondary outcomes

Below we describe the measurement instruments used in this study. The questionnaires, including a more detailed description and their psychometric properties can be found in the additional file on assessment instruments [see Additional file 1]. All questionnaires are administered in Dutch and all available Dutch versions of questionnaires are used.

#### Routine screening data

Routine screening data on somatic health, psychosocial functioning, substance use, medication use and PA is collected semi-annually in patients by trained HCPs.

Data on somatic health is used to calculate the QRISK3 score (i.e. HDL cholesterol, blood pressure, and BMI as calculated by dividing weight in kg by height in m^2^). Additionally, fasting glucose (mg/dL), triglycerides (mg/dL), weight (kg) and waist circumference are measured. Weight and waist circumference is measured to the first decimal (0.1 kg/cm) under the clothes at the level of the umbilicus (with the patient standing).

The Health of the Nations Outcomes Scale (HoNOS) is used to assess psychosocial functioning. The majority of patients will be measured with the HoNOS-12 [44]. The HoNOS 65 + [45] is used for ages 65 and above. The HoNOS-12 and HoNOS 65 + each have 12 items divided into four subscales.

Data on substance use is collected through routine questions about smoking (yes/no/stopped, if yes or stopped, how much on average; packyears are calculated), alcohol use (glasses per day, on average) and use of (soft)drugs (yes/no).

Medication related to cardiovascular health and diabetes, and psychotropic medication will be collected and converted into Defined Daily Dose (DDD) according the Anatomical Therapeutic Chemical Classification System from the World Health Organization (WHO) [46].

#### Lifestyle factors

##### *Physical Activity*

The Physical Activity Vital Sign (PaVs) is used to examine PA [47]. The PaVs is a 2-item questionnaire to assess whether patients meet the Dutch exercise guidelines for aerobic activity (yes/no). The PaVs for patients is obtained from routine screening. The PaVs is also assessed in HCPs through the online survey at each measurement.

The Simple Physical Activity Questionnaire (SIMPAQ) [48] is used to measure PA in addition to the PaVs. The SIMPAQ is a 5-item questionnaire which provides more detailed information about PA as compared to the PaVs and contains an additional measure on sedentary behaviour. The SIMPAQ is assessed in both patients and HCPs at each measurement.

##### *Diet and eating behaviour*

Diet will be assessed through a 24-h dietary recall (24HR) of all food and beverages consumed by patients. The Three Factor Eating Questionnaire-R18 (TFEQ-R18) is used to assess three domains of eating behaviour [49]. Diet and eating behaviour are assessed in patients at each measurement.

##### *Sleep and sleep medication*

The Scales for Outcomes in Parkinson’s disease sleep (SCOPA-Sleep) is used to evaluate sleep [50]. Additionally, information about the use of sleep medication is gathered. The SCOPA-Sleep is assessed in patients at each measurement.

#### Mental health outcomes

##### *Psychopathology*

The Brief Symptom Inventory (BSI) is used to measure symptoms of psychopathology [51]. The BSI consists of 53 items assessing nine symptom domains. The BSI is assessed in patients at each measurement.

##### *Quality of life*

Quality of life is measured with the EuroQol-5D (EQ-5D) [52] and the World Health Organisation Quality of Life-BREF (WHOQoL-BREF) [53]. The EQ-5D consists of five dimensions of health, with one item per dimension. The WHOQoL-BREF consists of four domains, measured through 24 items supplemented with two general health items. The EQ-5D is often used complementary to other quality of life instruments [54]. The EQ-5D and WHOQoL-BREF are assessed in patients at each measurement.

##### *Positive mental health*

The Mental Health Continuum – Short Form (MHC-SF) is used to examine positive mental health [55]. The MHC-SF measures positive mental health with 14 items, representing three domains of well-being. The MHC-SF is assessed in patients at each measurement.

#### Implementation factors

##### *Barriers and facilitators*

Barriers and facilitators regarding the implementation of MULTI + are assessed with the Measurement Instrument for Determinants of the Innovation (MIDI) [56]. This questionnaire consists of 29 items divided into four scales, which can be adapted to measure relevant constructs for the implementation. The MIDI can be used to identify which determinants influence the use of an innovation and improve or establish an implementation strategy. The MIDI is conducted in patients and HCPs, because both groups are considered end-users of MULTI + . The questions are adapted to fit the different end-users according to the manual. MIDIs are conducted in HCPs and patients before the implementation (Pre-MIDI) and after the implementation (Post-MIDI) of MULTI + . Thus, there are four different MIDIs available.

##### *Process evaluation*

To evaluate the implementation process and assess whether MULTI + is delivered as intended, a process evaluation will be conducted using the RE-AIM framework [57]. RE-AIM stands for the reach, effectiveness, adoption, implementation, and maintenance, and is often used to better interpret the effectiveness and implementation of an innovation. Data is continuously gathered from multiple sources, such as the facility services, patient files, questionnaires, and observations from research assistants.

##### *Adverse events*

Adverse events are investigated with an institutional program in which HCPs report and categorise incidents.

##### Motivation for behavioural regulation

Individual HCP factors, such as self-determined motivation, may influence the promotion of a healthy lifestyle in patients [58]. To assess motivation for behavioural regulation in exercise, the Behavioural Regulation in Exercise Questionnaire (BREQ-2) is used [59]. The BREQ-2 is comprised of 19 items divided into five scales. Furthermore, the BREQ-2 is used to devise a questionnaire assessing the motivation for Behavioural Regulation in Diet Questionnaire (BRDQ). All items used in the BREQ-2 assessing behavioural regulation in exercise were converted by changing the word “exercise” to “(eat a) healthy diet”. Both versions of the BREQ-2 are assessed in HCPs at each measurement.

##### Health Technology Assessment (HTA)

Cost-effectiveness is investigated through calculation of the costs of TAU as compared to costs of MULTI + . This social cost benefit analysis uses data from patient files to determine the costs of care used in the different conditions (TAU vs. MULTI +), and medication costs based on DDDs. Additionally, for the subgroup that answered questions regarding quality of life, we investigate impact on quality of life as opposed to costs. The total costs per measuring moment per patient is used as an outcome measure and expressed in euros.

### Research procedures

#### Recruitment

The implementation team informs the HCPs about the measurement procedures. Patients are informed about the study through HCPs and members of the implementation team (see Table 3 for participant timeline). Promotion materials will be used (such as videos, flyers and posters) to give information about the study.

**Table 3:** *Participant timeline*

Parameters		Time of Measurement			
		T0 (baseline)	T1 (six months)	T2 (twelve months)	T3 (eighteen months)
Informed consent^a^	PT & HCPs	x			
Demographics^a^	PT & HCPs	x			
Routine screening data^b^	EPR	x	x	x	x
Semi-structured interviews	PT	x	x	x	x
Online surveys	HCPs	x	x	x	x
Process evaluation	ongoing evaluation				
Adverse events					
Costs					

*PT* Patient, *HCPs* Health Care Professionals, *EPR* Electronic Patient Record

^a^Or at first enrolment in the study

^b^Data from routine screening is extracted from the EPR

#### Informed consent

Informed consent of HCPs is obtained online. With permission from the Medical Ethical Commission (METC), patients give verbal informed consent using a visual informed consent (VIC). A VIC is a visualisation of the goal and procedures of the study, and usage of personal data. The VIC makes information about the study easier to comprehend for patients. Researchers and research assistants are trained to conduct the informed consent procedure verbally. Patients will not be included in the study if they do not understand the information given, as deemed by HCPs or researchers.

#### Procedural considerations

The semi-structured interviews are conducted in a suitable place as determined by the researchers, HCPs and patients. If a patient is unable to adequately answer all questions, HCPs are asked for support when possible. If participating patients decease or discontinue inpatient treatment, they will not be included for further data collection. Data collected up until that point will be used for analyses. When patients withdraw consent, data already collected will be stored according to data protocol and Dutch law but will not be used for analyses or publications. After each measurement, 50 gift vouchers of 10 euros will be raffled among patients. HCPs will receive a small gift at ward level (such as a fruit basket or a water jug).

#### Study status

At the time of this manuscript submission data collection has commenced and final measurement is expected in August 2022.

### Statistical analyses

Within each cluster-intervention period (cluster I and TAU, cluster I and MULTI + , cluster II and TAU, cluster II and MULTI + etc.) we will analyse the QRISK3 of patients treated in that period. We select the last known measurements for each patient (longest exposure). For each of these measurements, the duration of exposure of patients in this cluster is calculated (date of last measurement minus day of entering this period). A proportion of patients will experience the switch in intervention during their stay. In such individuals, we will analyse both measurements reflecting the different conditions. The correlation of between subject measurements will be taken into account by adding a random effect for subjects. All QRISK3 data points will be analysed with mixed model analyses. We will analyse the logit transformed QRISK3 probabilities for similar reasons as in logistic regression, namely to avoid problems of estimating probabilities below 0 or above one and because it is more likely that the effect of treatment and other covariates on the outcome will be constant on the relative odds ratio scale.

The linear mixed model will contain: treatment (binary, 1/0 coding), cluster (categorical, two levels, dummy variables), time period (categorical, four levels, dummy variables), random effect for subject, the duration of exposure (continuous, restricted cubic splines with three knots); and the following potential confounding variables: age (continuous), key diagnostic subgroups (categorical) and illness severity (continuous).

Multiple imputation will be used to account for missing values. The same variables will be used in the imputation model as in the analysis model, extended with characteristics related to these variables. Multiple imputation based on chained equations will be used to generate 10 imputation sets (or more if the number of missing values is larger than expected). Results from imputed datasets will be pooled using Rubin’s rules.

Although MULTI + will be integrated into routine clinical care regardless of specific inpatient facility setting, we pragmatically set a minimum of 10 days exposure, assuming that we cannot expect an effect within a few days of hospitalization.

Explorative subgroup analyses will be performed to gain insight in possible treatment differences between the subgroups gender (categorical) age (continuous) and subjects with repeated-measures. Subgroup analyses will be performed consecutively by adding the main effect of these variables and their interaction to the main model specified above.

All analyses will be conducted with IBM SPSS statistics 25 and R version 4.0.4 or higher, with a 95% confidence interval (*p* < 0.05) and will be corrected for multiple outcomes/comparisons (Bonferroni correction) if necessary.

### Sample size

Our sample size considerations are based on demonstrating a difference in body weight between conditions. The reason is that we did not have any relevant data on changes in QRISK3 for this population, whereas body weight has been used in our previous research [32]. Furthermore, body weight is a key component of the QRISK3 and, given the nature of MULTI + , it is likely that other components of the QRISK3 will change in the same direction. Therefore, using body weight can be seen as a more robust and more conservative approach than using uncertain assumptions about changes in QRISK3.

Our research showed that 53.8% of the patients who participated in MULTI lost ≥ 5% of their initial bodyweight, as opposed to 16.3% of the patients who followed TAU. Given the broader implementation of MULTI + , with patients who spend less time in healthcare than those in the earlier study, we expect to find effects in a relative smaller number of patients in this study. Based on a small-scale follow-up, we will assume 32% of patients will lose ≥ 5% of their initial bodyweight instead of 53.8%. Because metabolic risk factors are a routinely measured outcome, we expect a conservative dropout rate of 10%. Because there is insufficient knowledge about the correlations between clusters, we will assume a correlation of 0.1. Based on these data, a power of 80%, a significance level of 5% (*p* < 0.05), three clusters, the minimal sample size should consist of 846 patients. Given the high patient turnover (n≈2000), this seems feasible.

### Data management

A data management protocol was created in which data entry, coding, security, and storage was recorded. Patient data is requested from the patient files through data management officers of the institution. Data is pseudonymized which only researchers within the implementation team have access to. Data is only accessible through two-way factor authorization. Data is stored and protected for 20 years within the institution, in line with national privacy laws, and will not be made publicly available.

## Discussion

This study protocol has been designed to evaluate the effectiveness and implementation of MULTI + in a specialist mental healthcare organisation with ~ 830 places of residence. To our knowledge, this will be the first large-scale study evaluating the long-term effects of a multidisciplinary, multicomponent approach aimed at improving lifestyle factors in routine inpatient clinical care. This study builds on previous research, which showed improvements on both physical and mental health outcomes in patients, and the need for more organisational support to continue and expand its implementation [32].

A reduction of ≥ 5% loss of body weight is associated with a relevant reduction in cardiovascular risk [60], which we therefore originally considered as the primary outcome of this study [61]. However, this was reconsidered after observing the high-patient turnover hindering individual follow-up and several other substantive considerations. Predominantly, this outcome measure does not account for other known risk factors for developing CVD, such as familial history, age, smoking behaviour, total cholesterol level, presence of mental illness and the use of atypical antipsychotics. By not taking these risk factors into account, the risk of CVD is often underestimated in people with MI and outcomes can be misinterpreted (e.g., lack of weight loss while reducing smoking behaviour) [62]. QRISK3 is a risk calculator that estimates individual risk of developing CVD in people between 25–84 years old, using multiple predictor variables that are especially relevant for people with MI. Therefore, the primary aim of this study is to investigate the difference in the mean QRISK3 score of patients receiving MULTI + compared to patients receiving TAU. To our knowledge there are no risk calculators suitable for ages under 25. Inpatients < 25 will therefore be excluded from this analysis, but will be included for secondary outcomes.

The study protocol should be viewed considering several limitations. A first limitation is the risk of missing data. For example, not all patients may be able to complete all questionnaires due to illness severity and cognitive deficits. Therefore, semi-structured interviews will be used to allow for more flexibility and clarification. Another reason for missing data could be patients refusing the administration of the routine screening. Moreover, some routine measurements are only assessed on clinical indication, and will not be present for each patient.

Another limitation is that implementation monitoring could be a challenge because of the large-scale, real-world conditions of the study. Furthermore, external events and organisation-wide changes may impact the effectiveness and implementation of MULTI + due to the non-controlled environment and may be difficult to account for.

Strengths of this study are that MULTI + allows for pragmatic implementation in real-world settings through co-design and tailoring of the 10 core components. We expect that this approach will increase long-term sustainability and can serve as a potential blueprint. Additionally, studying implementation factors will expand our knowledge on how to overcome the implementation gap and successfully implement a lifestyle intervention. Furthermore, we research a broad spectrum of both physical and mental health outcomes in inpatients with MI. This dual research focus on both implementation factors and health outcomes will expand our knowledge on the possible influences these factors have on one another. Ultimately, we aim to improve routine clinical care for inpatients with mental illness.

## Supplementary Information

**Additional file 1. **Assessment instruments MULTI+.docx. Detailed description of measurement instruments and their psychometric properties.

## Data Availability

Because our data is pseudonymised, it is therefore still considered personal data. Future data is only available upon reasonable request from the corresponding authors.

## Abbreviations

24HR: 24-Hour dietary recall
BMI: Body mass index
BRDQ: Behaviour regulation in diet questionnaire
BREQ-2: Behavioural regulation in exercise questionnaire-2
BSI: Brief symptom inventory
CM: Centimetres
CVD: Cardiovascular disease
DDD: Defined daily dose
EDC: Electronic data capture
EQ-5D: EuroQol-5d
HCPs: Health care professionals
HONOS: Health of the nation outcome scales
HTA: Health technology assessment
IC: Informed consent
KG: Kilograms
METC: Medical ethical committee
MG/DL: Milligrams per decilitre
MHC-SF: Mental health continuum-short form
MI: Mental illness
MIDI: Measurement instrument for determinants for innovations
MULTI +: Multidisciplinary lifestyle focused approach in the treatment of inpatients with mental illness
PA: Physical activity
PAVS: Physical activity vital sign
RCT: Randomized-controlled trial
SCOPA-SLEEP: Scales for outcomes in Parkinson’s disease-Sleep
SIMPAQ: Simple physical activity questionnaire
SMI: Severe mental illness
TAU: Treatment as usual
TFEQ-R18: Three factor eating questionnaire revised 18 items
VIC: Visual informed consent
WHO: World Health Organization
WHO-QOL: World health organization quality of life-bref
