# Supplementary material for: Effectiveness and implementation of a multidisciplinary lifestyle focused approach in the treatment of inpatients with mental illness (MULTI +): a stepped wedge study protocol
Source: BMC Psychiatry. 2022 Mar 31;22:230. doi: 10.1186/s12888-022-03801-w (PMC8973631; doi:10.1186/s12888-022-03801-w)
Supplement: Supplementary file 1 — Additional file 1. Assessment instruments MULTI+.docx. Detailed description of measurement instruments and their psychometric properties. [file 12888_2022_3801_MOESM1_ESM.docx]

Assessment instruments MULTI+

| ***Instrument Name, Developer, and Online availability*** | ***Instrument description*** | ***Psychometric Properties*** |
| --- | --- | --- |
| **Routine screening data** | | |
| 1. Health of the Nation Outcome Scales (HoNOS-12 and HoNOS 65+)   [HoNOS-12](https://www.trimbos.nl/docs/99a6c15d-6eaf-4073-bcd0-53bfcb997ac9.pdf)[1]  [HoNOS 65+](https://search.proquest.com/docview/2389376358?pq-origsite=gscholar&fromopenview=true)[2] | The HoNOS is a clinician rated instrument that measures psychosocial functioning. The HoNOS-12 is fit to use in adult population aged 16-64 years and the HoNOS 65+ is used for ages 65 and above. All items are scored on a five point Likert scale, ranging from 0 (no problem) to four (severe problem).  The HoNOS-12 consists of 12 items divided into four subscales: behavioural, impairment, symptoms, and social. The Dutch HoNOS-12 has an addendum consisting of an extra three items that can be used to measure medication adherence, treatment compliance and maniform disinhibition.  The HoNOS 65+ consists of 12 scales divided into four subscales: disability, psychiatric symptomatology, physical illness and psychosocial disturbance. Additionally, there are four items included in the addendum that can be used to measure medication adherence, lack of motivation for treatment, issues as a result of negative symptoms and maniform disinhibition. | Both versions of the HoNOS were assessed in Dutch adult and geriatric psychiatric populations. Total scale scores had reasonable to good reliability (ICC = .92), acceptable to good validity and are sensitive to measure change.[1, 3] Reliability increases the more is known about the patient. |
| **Lifestyle factors** | | |
| 1. [Physical Activity Vital Sign (PaVs](https://www.exerciseismedicine.org/assets/page_documents/EIM%20Physical%20Activity%20Vital%20Sign.pdf))[4] | The PaVs is a 2-item questionnaire, recommended as a brief way to routinely gain insight in physical activity levels.[4] The two questions are: *“On average how many days per week do you engage in moderate to vigorous physical activity like a brisk walk?”* and *“On those days, how many minutes on average do you engage in physical activity at this level?”* Based on this, it can be determined whether a patient meets the national physical activity guidelines for aerobic activity (yes = 1, no = 0). | The instrument is deemed a feasible method for assessing physical activity in a SMI population.[5] It showed moderate agreement with objective accelerometery (κ = .46, P < .001).[6] |
| 1. [Simple Physical activity Questionnaire (SIMPAQ](http://www.simpaq.org/))[7] | The SIMPAQ consists of five items (boxes). Participants are asked about time spent in bed (box 1), time sedentary, including naps (box 2), time spent walking (box 3), time spent exercising (box 4) and time spent in incidental activity, such as housekeeping (box 5). Time spent on these activities is asked on average over the past seven days. The total self-reported time spent on moderate-to vigorous physical activity (MVPA) can be calculated by adding box 4 and box 5.[7] | The SIMPAQ is a reliable and valid tool to assess physical activity in people with SMI. Reliability analyses indicate acceptable to good reliability with Spearman correlation coefficients between ρ = .63 and ρ = .76. Validity of moderate-to-vigorous physical activity was ρ = .25 for the entire sample and comparable to studies conducted in general population samples. The use of an alternative method for calculating sedentary behaviour is advised, since there was not enough evidence for the validity of the self-reported sedentary behaviour. In this study, the alternative method for calculating sedentary behaviour is used.[7] |
| 1. 24-hour dietary recall (24HR) | The 24HR is a retrospective assessment method for the purpose of a quick impression of foods consumed by an individual. For the purpose of this study, a 24-h recall was designed using the five-pass method. Foods and beverages consumed over the past 24 hours (from breakfast to breakfast) are collected, participants are asked about foods that may have been forgotten, time and occasion is recorded, detailed description is recorded and final questions are asked. | A 24HR dietary assessment can be used to describe the food intake of a population, examine relationships between variables or evaluate effectiveness of an intervention.[8] The five-pass method is a commonly used method in dietary assessment and is a simple way to reduce bias.[9] |
| 1. Three Factor Eating Questionnaire Revised 18-items ([TFEQ-R18](http://www.med.umich.edu/pdf/weight-management/TFEQ-r18.pdf))[10] | The Three Factor Eating Questionnaire Revised 18-items (TFEQ-R18) is a shorter version of the TFEQ, measuring eating behaviour. The TFEQ-R18 consists of 18 items on a 4-point Likert scale, measuring three domains: cognitive restraint, uncontrolled eating and emotional eating.[10]  For the purpose of this study the TFEQ-R18 was translated into Dutch, using forward translation and expert panel back-translation. | The TFEQ-18 has been used in various populations and has shown good reliability (α = .78 to .86) for all scales,[11] and is able to distinguish among different eating patterns.[12] |
| 1. Scales for Outcomes in Parkinson’s disease Sleep ([SCOPA-Sleep](https://www.lumc.nl/org/neurologie/research/park-dis/Scales/SCOPA-SLEEP/))[13] | The SCOPA-Sleep is developed to evaluate night-time sleep problems (NSP), daytime sleepiness (DS) and overall quality of sleep. The questionnaire enquires about the use of sleep medication and if so, which medication. All NSP and DS questions are scored on a 4-point Likert scale ranging from 0 (not at all/never) to three (a lot/often). Overall quality of sleep is scored on a 7-point Likert scale ranging from 0 (very well) to six (very badly).[13] | The SCOPA-SLEEP had good reliability on both NSP and DS (α = .88 and α = .91) and good construct validity in a Dutch sample of people with Parkinson disease. The scores on NSP, DS and sleep at night were highly correlated with already established validated measuring tools measuring these constructs.[13] |
| **Mental health outcomes** | | |
| 1. The Brief Symptom Inventory (BSI)[14]   Copyrighted forms and the BSI manual are available from the  publisher. | The BSI is a brief psychological self-report symptom scale measuring symptoms of psychopathology. It is an acceptable short alternative of the SCL-90-R. The BSI comprises 53 items that reflect nine symptom constructs of the SCL-90-R. Each item is rated on a 5-point scale of distress from 0 (not at all) to four (extremely).[14] | Internal consistency for the nine items range from α = .71 to α = .85 and the BSI is deemed a reliable measure over time.[14] Acceptable validity, sufficient test-retest reliability and good internal consistency with α > .80 on eight out of nine scales were found in a Dutch sample.[15] |
| 1. EuroQol-5D-5L ([EQ-5D](https://euroqol.org/))[16]   EuroQoL Group (1995).  The EQ-5D is available from the  publisher. | The EQ-5D is a generic instrument that consists of five dimensions of health, with one item per dimension: mobility, selfcare, usual activities, pain/discomfort, and anxiety/depression. Items are rated on a 3-point Likert scale ranging from 0 (no issues) to two (many issues). Additionally, a visual analogue scale (VAS) for overall health is included. The index scores are calculated ranging from 0 (worst quality of life) to one (perfect quality of life), using the Dutch value set based on time-trade-off considerations.[16] | The validity and reliability of the EQ-5D have been studied in patients with schizophrenia. Internal consistency was acceptable (α = .63). The EQ-5D was deemed a valid and reasonably reliable instrument.[17] |
| 1. [World Health Organisation Quality of Life (WHOQoL-BREF)](https://www.who.int/toolkits/whoqol/whoqol-bref)[18] | The four WHOQoL-BREF domains are measured through 24 domain-specific items: physical health, psychological, social relations and environment and two general health items.[19] Item scores have various options but always range from one to five, such as very poor to very good, or not at all to extremely, and are converted to domain scores (range from four to 20) based on the WHO guidelines.[18] | The WHOQoL-BREF is investigated within people diagnosed with a psychiatric disorder and showed satisfactory to good internal consistency (α = .66 to α = .80), as well as in a schizophrenic patient population and showed good content and construct validity.[19] |
| 1. [Mental Health Continuum - Short Form](https://www.aacu.org/sites/default/files/MHC-SFEnglish.pdf) (MHC-SF)[20] | The original MHC-SF was derived from the Mental Health Continuum Long Form (MHC-LF) and first investigated in a South African sample [20]. The MHC-SF measures positive mental health and consists of 14 items, representing feelings of well-being: emotional well-being, psychological well-being and social well‐being. Participants rate the frequency of these feelings in the past month on a 6-point Likert scale ranging from never to every day. | Good to adequate internal reliability was found for the total score (α = .89) and subscales (α = .74 to α = .83) of the Dutch version of the MHC-SF. The test-retest reliability was found to be moderate, indicating that the MHC-SF is sensitive to changes over time. Additionally, good convergent validity was found, suggesting that the Dutch MHC-SF is a valid measurement instrument.[21] |
| **Implementation factors** | | |
| 1. [Measurement Instrument for Determinants for Innovations (MIDI)](https://www.tno.nl/media/6077/fleuren_et_al_midi_measurement_instrument.pdf)[22] | The MIDI was developed as a short and generic instrument to help improve the understanding of critical determinants regarding the implementation of innovations.[22] The MIDI is comprised of 29 items, divided into four scales measuring determinants associated with: innovations, the user, the organization and the socio-political context. Items are scored on a 5-point Likert scale ranging from one (totally disagree) to five (totally agree). Researchers can decide which determinants to measure, and adapt the questions so they are suitable for the innovation. Since this study is being conducted in a single organisation, the socio-political context is not assessed. | N/A |
| 1. [Behavioral Regulation in Exercise Questionnaire – 2 (BREQ-2)](http://exercise-motivation.bangor.ac.uk/breq/breqdown.php)[23] | The BREQ-2[23] was modified based on the BREQ[24] and includes an extra assessment of amotivation. The BREQ-2 is comprised of 19 items, divided into five scales measuring: amotivation, external, identified, introjected and intrinsic regulations. Questions are scored on a 5-point Likert scale ranging from 0 (not true for me) to four (very true for me).  For the purpose of this study, two items (six and 16) were changed in an attempt to better assess the relationship between behavioural regulation and the MULTI+, which is initiated by the employer. Item six was changed from: “I take part in exercise because my friends/family/partner say I should” to “I take part in exercise because my employer says I should” and item 16 was changed from “I feel under pressure from my friends/family to exercise” to “I feel under pressure from my employer to exercise”. | The BREQ-2 was subjected to confirmatory factor analyses and excellent model fit was found. The BREQ-2 has acceptable internal consistency and was found to be a reliable instrument with α = .73 to .86.[23] |
| 1. Behavioural Regulation in Diet Questionnaire (BRDQ) | Based on the BREQ-2 as used in this study, the researchers devised a behavioural regulation diet questionnaire. All mentioning of “exercise” in the BREQ-2 has been replaced by referring to “(eat a) healthy diet”. | N/A |

SMI: severe mental illness.

1. Mulder CL, Staring ABP, Loos J, et al. De Health of the Nation Outcome Scales (HONOS) als instrument voor 'routine outcome assessment'. [The Health of the Nation Outcome Scales (HONOS) in Dutch translation as an instrument for Routine Outcome Assessment]. *Tijdschrift voor Psychiatrie* 2004;46:273-84.

2. Burns A, Beevor A, Lelliott P, et al. Health of the Nation Outcome Scales for Elderly People (HoNOS 65+): Glossary for HoNOS 65+ score sheet. *British Journal of Psychiatry* 1999;174:435-8 doi:10.1192/bjp.174.5.435.

3. Broersma TW, Sytema S. De Health of the Nation Outcome Scale als effectmaat in de ouderenpsychiatrie. *Tijdschrift voor Gerontologie en Geriatrie* 2012;2010:13-8 doi:10.1007/s12439-010-0003-0.

4. Greenwood JL, Joy EA, Stanford JB. The Physical Activity Vital Sign: a primary care tool to guide counseling for obesity. *Journal of Physical Activity and Health* 2010;7:571-6 doi:10.1123/jpah.7.5.571.

5. Vancampfort D, Stubbs B, Probst M, et al. Physical activity as a vital sign in patients with schizophrenia: evidence and clinical recommendations. *Schizophrenia Research* 2016;170:336-40 doi:10.1016/j.schres.2016.01.001.

6. Ball TJ. Validity of a self-reported" vital sign" for physical activity in adults of primary healthcare: The University of Utah: 2014.

7. Rosenbaum S, Morell R, Abdel-Baki A, et al. Assessing physical activity in people with mental illness: 23-country reliability and validity of the simple physical activity questionnaire (SIMPAQ). *BMC Psychiatry* 2020;20:108 doi:10.1186/s12888-020-2473-0.

8. Vereecken C, Covents M, Sichert-Hellert W, et al. Development and evaluation of a self-administered computerized 24-h dietary recall method for adolescents in Europe. *International journal of obesity* 2008;32:S26-S34 doi:10.1038/ijo.2008.180.

9. Moshfegh AJ, Rhodes DG, Baer DJ, et al. The US Department of Agriculture Automated Multiple-Pass Method reduces bias in the collection of energy intakes. *The American Journal of Clinical Nutrition* 2008;88:324-32 doi:10.1093/ajcn/88.2.324.

10. Karlsson J, Persson L-O, Sjöström L, et al. Psychometric properties and factor structure of the Three-Factor Eating Questionnaire (TFEQ) in obese men and women. Results from the Swedish Obese Subjects (SOS) study. *International Journal of Obesity* 2000;24:1715-25 doi:10.1038/sj.ijo.0801442.

11. Brytek-Matera A, Rogoza R, Czepczor-Bernat K. The Three-Factor Eating Questionnaire-R18 Polish version: factor structure analysis among normal weight and obese adult women. *Archives of Psychiatry and Psychotherapy* 2017;3:81-90 doi:10.12740/APP/76342.

12. De Lauzon B, Romon M, Deschamps V, et al. The Fleurbaix Laventie Ville Sante (FLVS) study group: the three-factor eating questionnaire-R18 is able to distinguish among different eating patterns in a general population. *The Journal of Nutrition* 2004;2380:2372-004 doi:10.1093/jn/134.9.2372.

13. Marinus J, Visser M, van Hilten JJ, et al. Assessment of Sleep and Sleepiness in Parkinson Disease. *Sleep* 2003;26:1049-54 doi:10.1093/sleep/26.8.1049.

14. Derogatis LR, Melisaratos N. The Brief Symptom Inventory: an introductory report. *Psychological Medicine* 1983;13:595-605 doi:10.1017/S0033291700048017.

15. De Beurs E, Zitman F. De Brief Symptom Inventory (BSI): De betrouwbaarheid en validiteit van een handzaam alternatief voor de SCL-90. *Maandblad Geestelijke Volksgezondheid* 2005;61:120-41.

16. Rabin R, Oemar M, Oppe M, et al. EQ-5D-5L user guide. *Basic information on how to use the EQ-5D-5L instrument Rotterdam: EuroQol Group* 2011;22.

17. Pitkänen A, Välimäki M, Endicott J, et al. Assessing quality of life in patients with schizophrenia in an acute psychiatric setting: reliability, validity and feasibility of the EQ-5D and the Q-LES-Q. *Nordic Journal of Psychiatry* 2012;66:19-25 doi:10.3109/08039488.2011.593099.

18. WHO. WHOQOL-BREF: introduction, administration, scoring and generic version of the assessment: field trial version. WHO; 1996.

19. Trompenaars FJ, Masthoff ED, Van Heck GL, et al. Content validity, construct validity, and reliability of the WHOQOL-Bref in a population of Dutch adult psychiatric outpatients. *Quality of Life Research* 2005;14:151-60 doi:10.1007/s11136-004-0787-x.

20. Keyes CL, Wissing M, Potgieter JP, et al. Evaluation of the mental health continuum–short form (MHC–SF) in setswana‐speaking South Africans. *Clinical Psychology & Psychotherapy* 2008;15:181-92 doi:10.1002/cpp.572.

21. Lamers SM, Westerhof GJ, Bohlmeijer ET, et al. Evaluating the psychometric properties of the mental health continuum‐short form (MHC‐SF). *Journal of Clinical Psychology* 2011;67:99-110 doi:10.1002/jclp.20741.

22. Fleuren MA, Paulussen TG, Van Dommelen P, et al. Towards a measurement instrument for determinants of innovations. *International Journal for Quality in Health Care* 2014;26:501-10 doi:10.1093/intqhc/mzu060.

23. Markland D, Tobin V. A modification to the behavioural regulation in exercise questionnaire to include an assessment of amotivation. *Journal of Sport and Exercise Psychology* 2004;26:191-6 doi:10.1123/jsep.26.2.191.

24. Mullan E, Markland D, Ingledew DK. A graded conceptualisation of self-determination in the regulation of exercise behaviour: Development of a measure using confirmatory factor analytic procedures. *Personality and Individual Differences* 1997;23:745-52 doi:10.1016/S0191-8869(97)00107-4.
